# Supplementary material for: Hypoxia tolerance, but not low pH tolerance, is associated with a latitudinal cline across populations of Tigriopus californicus
Source: PLoS One. 2022 Oct 27;17(10):e0276635. doi: 10.1371/journal.pone.0276635 (PMC9612455; doi:10.1371/journal.pone.0276635)
Supplement: S1 Table — Pairwise distances were computed in MEGA [102] using the p-distance method. Sequences for AB, BB, SC, SD, and SS were downloaded from Genbank (accession numbers included). The sequence for FHL was determined from genomic sequencing. Accession number to be determined. (DOCX) [file pone.0276635.s001.docx]

S1 Table

|  | SS | SC | BB | AB | SD | FHL |
| --- | --- | --- | --- | --- | --- | --- |
| SS  (GQ140992.1) | -- |  |  |  |  |  |
| SC  (GQ140977.1) | 0.198 | -- |  |  |  |  |
| BB  (GQ358922.1) | 0.206 | 0.153 | -- |  |  |  |
| AB  (GQ140855.1) | 0.222 | 0.206 | 0.208 | -- |  |  |
| SD  (GQ140645.1) | 0.214 | 0.204 | 0.218 | 0.2 | -- |  |
| FHL | 0.22 | 0.179 | 0.151 | 0.197 | 0.22 | -- |
