# Supplementary material for: Hypoxia tolerance, but not low pH tolerance, is associated with a latitudinal cline across populations of Tigriopus californicus
Source: PLoS One. 2022 Oct 27;17(10):e0276635. doi: 10.1371/journal.pone.0276635 (PMC9612455; doi:10.1371/journal.pone.0276635)
Supplement: S2 Table — (DOCX) [file pone.0276635.s002.docx]

**S2 Table *Tigriopus californicus* collection locations, dates, and sample sizes**.

| Population | Abbreviation | Latitude,  Longitude | Date of Collection (MM/DD/YY) | Sample Size: Hypoxia (sex)  Low pH (sex) |
| --- | --- | --- | --- | --- |
| (1) Friday Harbor Laboratories | FHL | 48.449974,  -122.963021 | 4/3/18 | 114 (57 f, 57 m)  80 (40 f, 40 m) |
| (2) Bodega Marine Lab | BB | 38.305133,  -123.065556 | 7/9/13 | 119 (60 f, 59 m)  79 (40 f, 39 m) |
| (3) Santa Cruz | SC | 36.949617,  -122.046940 | 11/21/17 | 120 (60 f, 60 m)  80 (40 f, 40 m) |
| (4) San Simeon | SS | 35.581633,  -121.121111 | 9/27/04 | 114 (55 f, 59 m)  79 (40 f, 39 m) |
| (5) Abalone Cove | AB | 33.740733,  -118.377778 | 5/24/11 | 116 (60 f, 56 m)  77 (38 f, 39 m) |
| (6) San Diego | SD | 32.745646,  -117.255036 | 11/22/17 | 113 (55 f, 58 m)  77 (39 f, 38 m) |
