# Supplementary material for: Hypoxia tolerance, but not low pH tolerance, is associated with a latitudinal cline across populations of Tigriopus californicus
Source: PLoS One. 2022 Oct 27;17(10):e0276635. doi: 10.1371/journal.pone.0276635 (PMC9612455; doi:10.1371/journal.pone.0276635)
Supplement: S3 Table — Low oxygen conditions were obtained by bubbling nitrogen gas into the water bath and monitoring swimming as described in the Hypoxia and Low pH Assay protocol. Low pH conditions were obtained by bubbling carbon dioxide gas into the water bath and monitoring swimming as described in the Hypoxia and Low pH Assay protocol. High pH conditions were obtained by adding concentrated sodium hydroxide solution to sea water and measuring swimming at the end of 24 h of exposure. (DOCX) [file pone.0276635.s003.docx]

S3 Table

| **Duration** | **DO** | **pH** | **Percent Swimming**  **(n = sample size)** |
| --- | --- | --- | --- |
| 6 Hours | 0.00 | -- | 67% (n = 12) |
| 50 Hours | 0.1 | -- | 75% (n = 32) |
| 16 Hours | -- | 5.5 | 91% (n = 120) |
| 1 Hour | -- | 5.0 | >1% (n = 130) |
| 24 Hours | -- | 10.2 | 93% (n = 60) |
