# Supplementary material for: Hypoxia tolerance, but not low pH tolerance, is associated with a latitudinal cline across populations of Tigriopus californicus
Source: PLoS One. 2022 Oct 27;17(10):e0276635. doi: 10.1371/journal.pone.0276635 (PMC9612455; doi:10.1371/journal.pone.0276635)
Supplement: S4 Table — Measurements from five tide pools at each of four latitudes along the California coastline were averaged by time and location. Standard deviations are given in parentheses. Measurements are divided by morning, afternoon, or evening. Locations are ordered from northernmost (BB) to southernmost (SD). (DOCX) [file pone.0276635.s004.docx]

**S4 Table Observations of tide pool conditions containing *T. californicus* during August 2021.**

| Time | pH | DO in mg/L |
| --- | --- | --- |
| Morning | *BB* 7.98 (±0.31)  *SC* 8.02 (±0.40)  *AB* 8.04 (±0.11)  *SD* 8.09 (±0.46) | *BB* 5.4 (±2.1)  *SC* 3.1 (±0.6)  *AB* 3.6 (±0.9)  *SD* 4.2 (±2.6) |
| Afternoon | *BB* (unavailable)  *SC* 8.26 (±0.37)  *AB* 8.43 (±0.14)  *SD* 8.79 (±0.38) | *BB* 11.8 (±0.9)  *SC* 9.1 (±2.9)  *AB* 7.9 (±1.0)  *SD* 8.8 (2.2) |
| Evening | *BB* 9.21 (±0.13)  *SC* 8.51 (±0.41)  *AB* 8.67 (±0.19) *SD* 8.87 (±0.36) | *BB* 9.1 (±1.5) *SC* 8.5 (±4.1)  *AB* 7.8 (±1.1)  *SD* 6.7 (±1.5) |
