# Supplementary material for: Hypoxia tolerance, but not low pH tolerance, is associated with a latitudinal cline across populations of Tigriopus californicus
Source: PLoS One. 2022 Oct 27;17(10):e0276635. doi: 10.1371/journal.pone.0276635 (PMC9612455; doi:10.1371/journal.pone.0276635)
Supplement: S5 Table — Significant p-values are written in bold. Regression coefficients are written with 95% confidence intervals. Importance is the weighted contribution of a predictor across all models compared. Importance values greater than 0.50 are noted with an asterisk (*). (DOCX) [file pone.0276635.s005.docx]

| Hypoxia Model Predictor  df = 14 | *p* | Regression Coefficients (β) ± 95% Confidence Interval | Importance (I) |
| --- | --- | --- | --- |
| Latitude | **<0.001** | 7.82226 ± 3.0819144 | 1.00* |
| Sex (male) | 0.545342 | -0.2156621 ± 0.95505 | 0.63* |
| Length | **<0.001** | -1.435299 ± 0.7597687 | 1.00* |
| Collection Year | **<0.001** | -5.127622 ± 2.16483 | 1.00* |
| Latitude × Sex | 0.704402 | 0.1215641 ± 0.376752 | 0.25 |
| Latitude × Length | 0.713292 | 0.07461517 ± 0.42084307 | 0.33 |
| Latitude × Year | **<0.001** | -6.259887 ± 2.5606168 | 1.00* |
| Sex × Length | 0.822760 | 0.0481855 ± 0.6275338 | 0.20 |
| Sex × Year | 0.913274 | -0.01750043 ± 1.18408507 | 0.17 |
| Length × Year | 0.935785 | 0.01313413 ± 0.63216393 | 0.26 |
