# Supplementary material for: Hypoxia tolerance, but not low pH tolerance, is associated with a latitudinal cline across populations of Tigriopus californicus
Source: PLoS One. 2022 Oct 27;17(10):e0276635. doi: 10.1371/journal.pone.0276635 (PMC9612455; doi:10.1371/journal.pone.0276635)
Supplement: S6 Table — Significant p-values are written in bold, and regression coefficients are written with 95% confidence intervals. Importance is the weighted contribution of a predictor across all models compared. Importance values greater than 0.50 are noted with an asterisk (*). (DOCX) [file pone.0276635.s006.docx]

| pH Model Predictor  df = 8 | *p* | Regression Coefficient (β) ± 95% Confidence Interval | Importance (I) |
| --- | --- | --- | --- |
| Latitude | 0.9777 | -0.01100642 ± 1.56668978 | 0.27 |
| Sex (male) | **0.0404** | -1.614681 ± 1.4749847 | 0.90* |
| Length | 0.9772 | 0.008333252 ± 1.132061952 | 0.26 |
| Collection Year | 0.4828 | 0.4731268 ± 0.829561 | 0.49 |
