# Supplementary figures and images for: Hypoxia tolerance, but not low pH tolerance, is associated with a latitudinal cline across populations of Tigriopus californicus
Source: PLoS One. 2022 Oct 27;17(10):e0276635. doi: 10.1371/journal.pone.0276635 (PMC9612455; doi:10.1371/journal.pone.0276635)

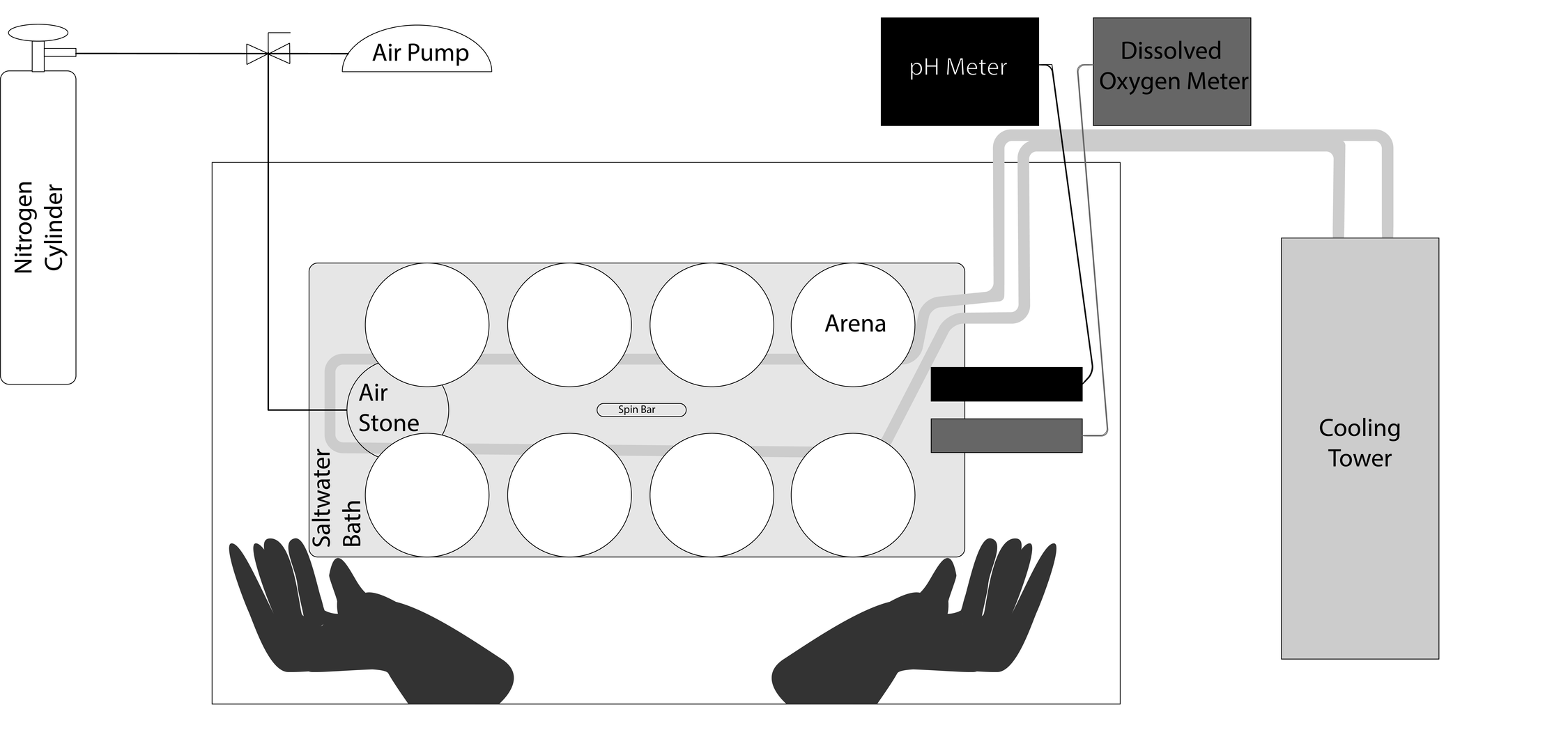

Supplement: S1 Fig — The glove box was constructed of ¼” plexiglass, laser cut to measure 26 in (w) x 14 in (l) x 18 in (d) with a side window held with latch clamps. Seams and cracks were sealed with epoxy. Rubber kitchen gloves were held by hose clamps attached to 3 in to 4 in sewer drain adapters, tightly fitted and sealed with epoxy to the front of the glovebox. Laser cutter blueprints for the box are included on Dryad. (TIF) [file pone.0276635.s007.tif]

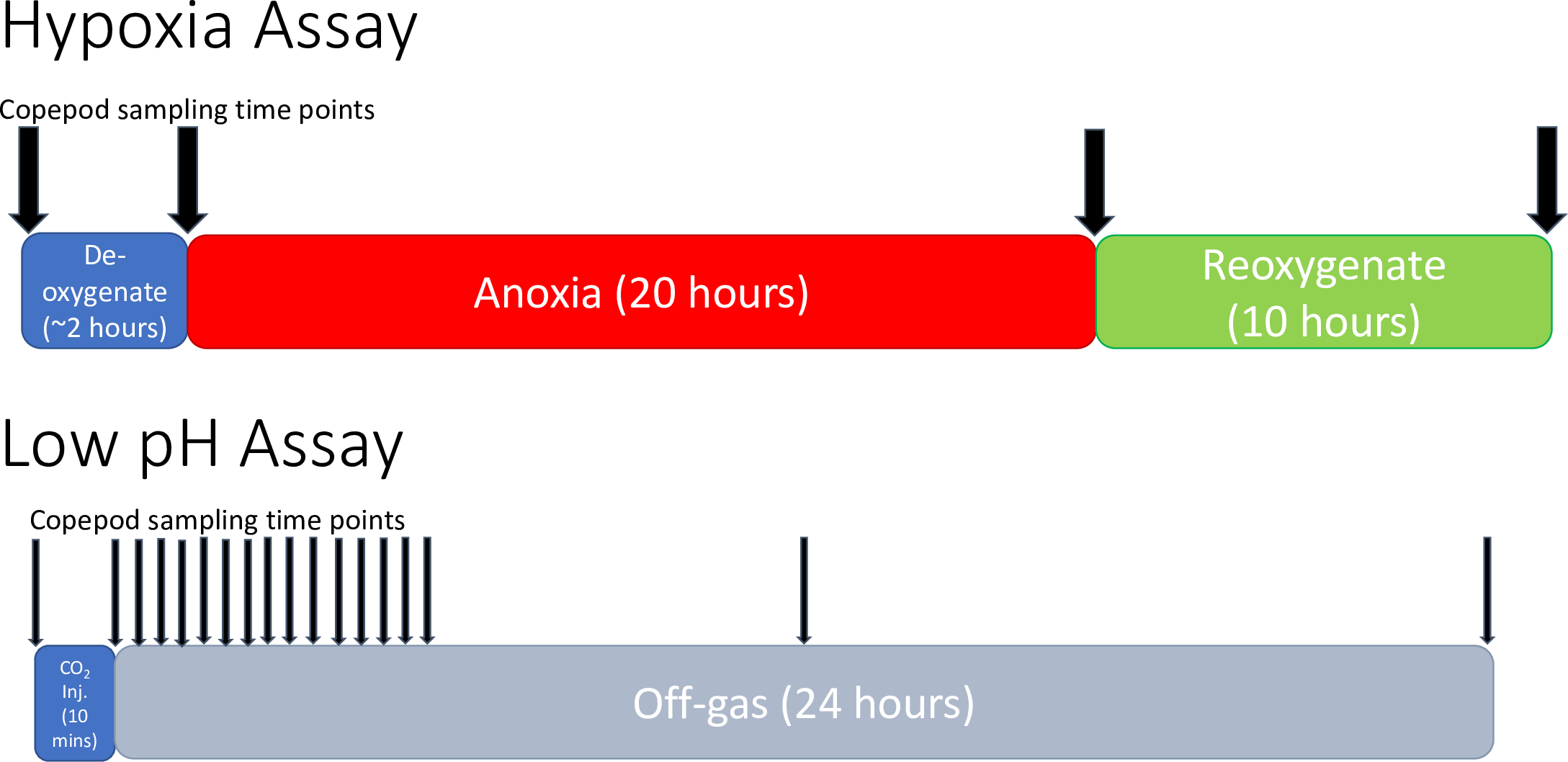

Supplement: S2 Fig — Black arrows indicate sampling time points. (TIF) [file pone.0276635.s008.tif]

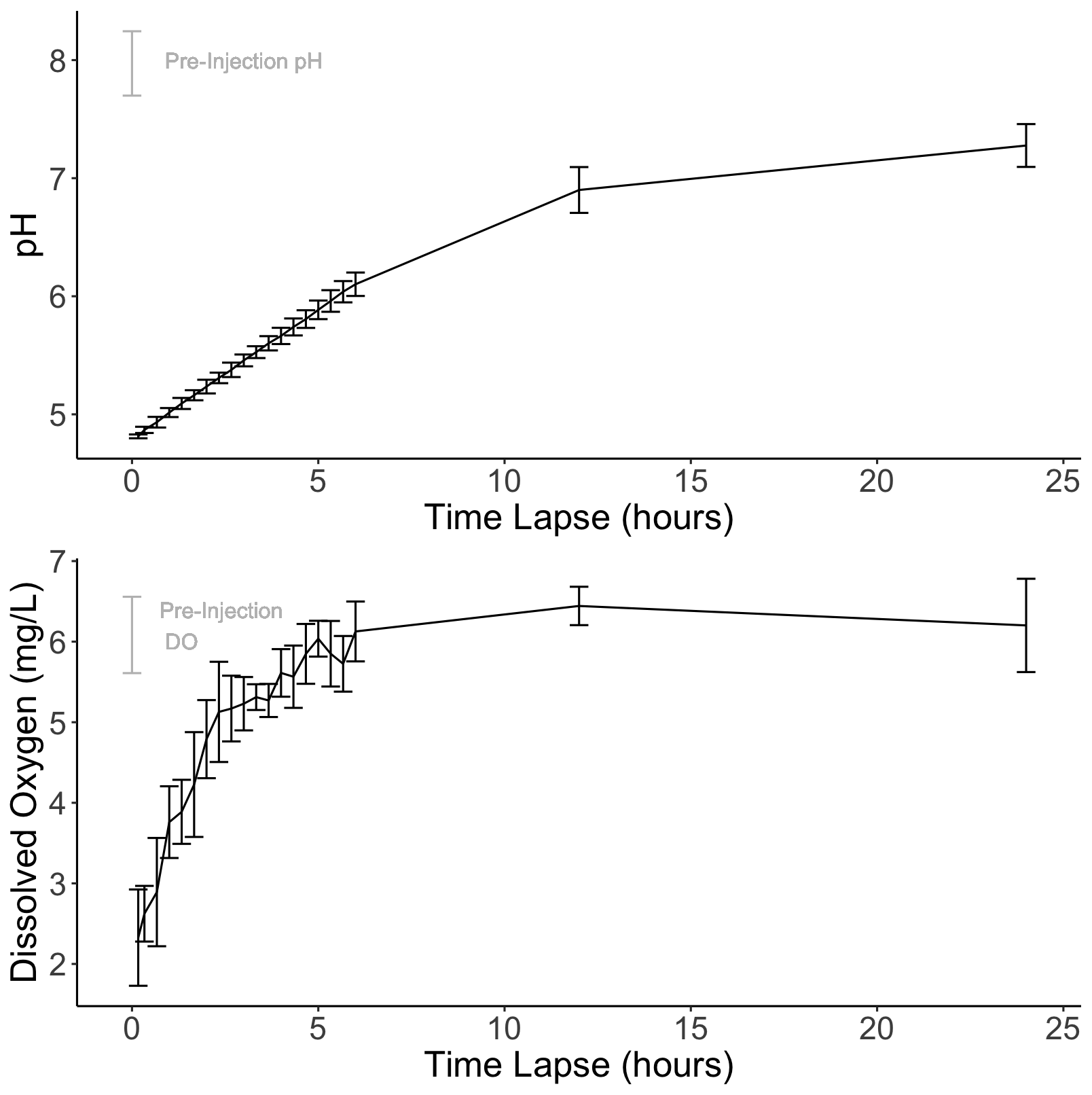

Supplement: S3 Fig — Mean pH (top) and DO (bottom) values during low pH assays are displayed with 1 SD bars. The pH and DO of the water bath prior to carbon dioxide injection is indicated in gray. (TIF) [file pone.0276635.s009.tif]

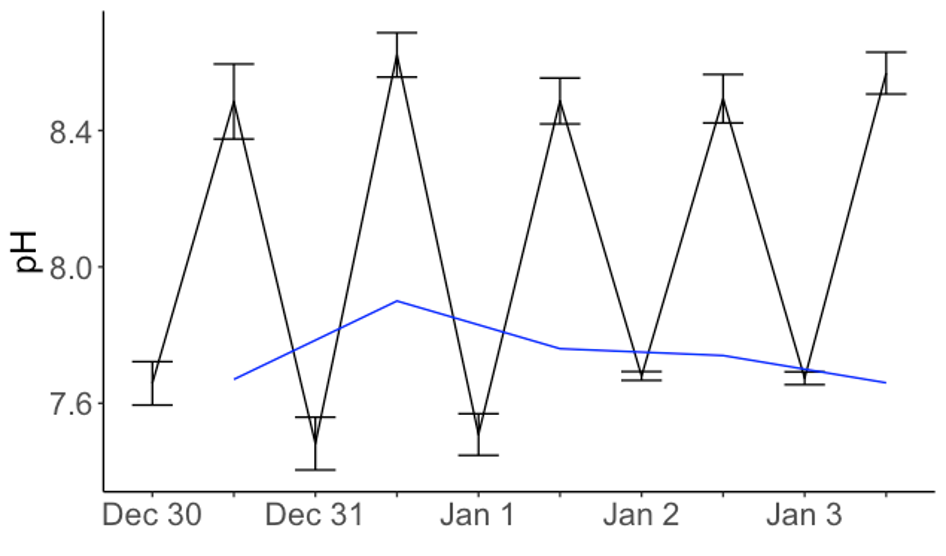

Supplement: S4 Fig — All pH measurements were taken with a handheld pH meter (Oakton Waterproof pH Testr® 30) in the winter of 2017–2018. Rockpool measurements were taken at sunrise and sunset, while ocean measurements were only taken at sunset. The blackline represents rockpool values whereas the blue line represents ocean values. The values of 6 rockpools were averaged. Bars are standard errors of the mean. (TIF) [file pone.0276635.s010.tif]
