# Supplementary material for: Hypoxia tolerance, but not low pH tolerance, is associated with a latitudinal cline across populations of Tigriopus californicus
Source: PLoS One. 2022 Oct 27;17(10):e0276635. doi: 10.1371/journal.pone.0276635 (PMC9612455; doi:10.1371/journal.pone.0276635)
Supplement: Attachment — Submitted filename: Review_Deconinck_etal.docx [file pone.0276635.s011.docx]

General Comments:

This manuscript looks at effects of body size, latitude (collection location), and sex on hypoxia tolerance and low pH tolerance in the copepod *Tigriopus californicus*. This paper examined how copepod cultures from six locations ranging from San Diego, CA to Friday Harbor Labs in Washington survive when exposed to acute exposures of low oxygen concentration and low pH. The authors hypothesized 1) that both stressors co-occuring would yield population-specific tolerance to either stressor, 2) that hypoxia and pH tolerance would inversely correlate with latitude, and 3) one of the evaluated effects would predict tolerance. The authors found that while latitude and length are predictors of performance with respect to hypoxia tolerance, only sex is a predictor for pH tolerance. Patterns of hypoxia tolerance with respect to latitude are converse to the authors expectations and may reflect duration of lab rearing rather availability of oxygen.

This is a very interesting study. I have few comments on the nature of this work. It is well designed, executed and analyzed. My only criticism would be the interpretation. There is nothing wrong with how the data is interpreted as it is, but it could benefit from more explanation or a deeper dive into why the authors believe they observed the patterns they did, particularly when considering the hypoxia tolerance pattern with latitude. I recommend the authors consider a few more ideas as to why hypoxia tolerance increases with latitude rather than decreases. Is there an evolutionary pattern that may be at play? Is the tolerance strictly plastic?

Specific comments:

Lines 49-50, 308, 326-327: Fix references to match citation style.

Lines 204-206: “Importance” comes up a lot in this manuscript. How does one evaluate what the critical level of importance is? In other words, how important is “important”? Does a value >0.5 suggest that it is somehow not random?

Table 2 – A figure depicting the temperature, DO, and pH changes across a day for each location would be easier to interpret. If possible, the authors should consider making one.

Lines 249-250: “…although the latter was opposed by the effect of latitude.” What does it mean for one effect to oppose another? Is this meant to represent an antagonistic relationship?

Lines 310-315: So, hypoxia tolerance is a physical attribute and pH tolerance is a physiological one? This seems interesting. I suggest the authors discuss how both tolerance systems may involve physical and/or physiological mechanisms. For instance, the upregulation of oxidative stress proteins does involve certain reactive oxygen species that can be accumulated from hypoxia, not just low pH. The authors should expand on other stress tolerance mechanisms that are different for each of the stressors discussed in the manuscript.

Lines 324-335: This is very interesting and deserves more discussion. Please include more detail as to what the “temporal” correlations are. Are they across years? Or across the day?
