# Supplementary material for: Hypoxia tolerance, but not low pH tolerance, is associated with a latitudinal cline across populations of Tigriopus californicus
Source: PLoS One. 2022 Oct 27;17(10):e0276635. doi: 10.1371/journal.pone.0276635 (PMC9612455; doi:10.1371/journal.pone.0276635)
Supplement: Attachment — Submitted filename: PONE_review.docx [file pone.0276635.s012.docx]

Latitudinal cline in hypoxia tolerance does not result in correlated acid tolerance in Tigriopus californicus

This paper aims to determine if there is a correlation between low oxygen and low pH tolerance in populations of copepods from a broad latitudinal range. The topic is of great importance to the field and a better understanding of how the temporal variability of these two stressors impacts marine animals is needed. Including latitude and population-level responses is especially helpful, and difficult to do, making this contribution important. However, there are some issues with the current draft that should be addressed before publication. I hope my comments below are clear and helpful.

- The environmental data presented did not include continuous or night-time values. This is when you would expect the DO and pH minima to occur, thus linkages between the experimental design and field data are weak, and are the assumptions based on them. There isn’t much information about conditions as they relate to the latitudinal cline either.
- If I understood correctly, the low pH trials are confounded by co-occurring low O2. Even if the O2 levels were not at the experimental target (0.5 mg/L I think), 2 mg/L O2 during the low pH trials is still quite low (and hypoxic as this paper defines it!). To me, this makes it very difficult to say that there is ‘no evidence of correlation in tolerance for these two stressors’ Line 308. This should be discussed and addressed, and the correlation, and its interpretation, between the low O2 and pH trials should be reconsidered.
- Additionally, I found it misleading to discuss correlation between the low-O2 and low pH traits when they were not measured together (but separate single-stressor studies). I think that could be fixed by carefully fixing the text so that there is no confusion that a multiple stressor study was not performed, but single stressor trials. It also brings up the question of why the authors didn’t test these stressors together? There are many examples of multiple stressors (together) having very different, and important physiological impacts compared to single stressors.
- It would be helpful if figures were reworked to help the reader to see the significant impact hypoxia and low pH (separately) have across populations/sites. A lot of the supplemental material was important and could/should be condensed and added to the main MS so the reader can more easily follow along with all the pertinent information readily available (e.g., a map that shows where the sites are and how they are environmentally distinct).
- It might be helpful to discuss the results from the low pH trial and the low oxygen trial separately and put less emphasis on the correlation analysis.

Minor points:

Line 26 – both stressors when tested together? Will be helpful to differentiate single vs combined stress responses throughout the text

Citation style format might be correct for journal, but seems to vary between numbers (13) and names (Edmands, 2018) throughout the text.

Line 57 add references for this. Also, specify that these multiple studies are (or are not) linking temperature, hypoxia and acid tolerance together as an adaptation. It’s confusing because I think you are trying to say that there are not studies on this topic (which I agree with and is the point you should emphasize -because your study is one of the first to test this). The first sentence of this paragraph doesn’t really say what I think you are trying to say here.

‘Low pH’ is a better word to use than ‘acid’ – I would change this and be consistent throughout. As is, there is a mixture of terminology used.

Also, define ‘hypoxia’ is border-line jargon without an exact definition, make sure this is defined once and used in that context throughout.

Line 63- is Pc Pcrit?

Line 88 – this is implies that both stressors are tested together, not singularly, this should be clarified

Line 92-98- It would be helpful to add a map of all the locations, how far away from each other are these sites? The field measurement section here also could benefit from a figure showing the diurnal flux at the different sites, is it approx. the same magnitude along the gradient?

The field data should be shown in the main text, maybe on the map, to highlight differences between sites

Line 109- where these copepod cultures all being kept at the Bodega Marine Lab?

Line 114- up until now I thought the study was a multiple stressor study, not two single stressor studies. It would be helpful to ensure the experimental design is clear right away (in the intro). Also, was a multiple stressor (i.e., combined stressor) treatment attempted? If not, why? There can be different responses (synergistic, additive, antagonistic) when the stressors occur in combination, so some discussion on this missing element in the study would be beneficial.

Table 1 could be converted into a map, and sent to the supplemental materials

Line 117- where did experiments take place?

Line 148- experimental design. It would be helpful if the authors briefly mentioned the experimental design earlier in the intro and methods. Also, please specify earlier what the target DO/pH levels were.

How often were DO measurements were made during the trials (same for pH?), and what the variability in both pH and DO during these trials?

How was the oxygen kept stable?

It is intriguing to me that the pH went up during these trials, what was the starting pH? Why would this be?

Line 152- can the authors elaborate on this experiment and how/why they chose the pH and DO values for the current study? It would be very helpful to be more specific about the exploratory experiments -this is interesting. – i.e., ‘75% of tested copepods were able to swim after xx hour exposure to oxygen concentrations of xx mg/L, therefore we chose to xx’

The pH values seem very high to me. Is this normal?

Line 157- Please add the accuracy/precision of this DO probe and describe how it waw calibrated. Was this done with a 0-point calibration solution?

Line 156- hypoxia was maintained for 20 hours- does this include the ~2 hours of ramping (when the nitrogen was turned on?)

Line 163- again, more details would be very helpful about the previous experiments

Line 161 – so the animals were exposed to 24 of low pH and 20 hours of low DO?

Line 166- was this the same for the DO trial?

Line 182- how many copepods were not recovered? Why was this? Please put a number as to how many were lost. This is a concern if they were escaping from their cages.

Figure S5 and S6- It would be useful to add time to these plots, to help identify what time of day you are referring to. Sunset and sunrise? What about noon? S6 is missing DO units. Also, would be good to have standard deviation for both of these to make comparisons easier.

Line 219- again, it would be nice to have a map with this information in it, showing the variation in pH and DO in tidepools along the latitudinal gradient. It would be much easier to understand if Table 2, and Fig S5 and S6 were combined into one figure, in the main MS.

Line 231- what about night-time values? There are a lot of studies and data showing the pH and oxygen minima occur at night due to respiration. This is missing, and an important aspect to making claims such as this about DO, pH and latitudinal trends. Authors should take care in making interpretations from their measurements, or add additional data from other sources to help.

Line 236 – Please clarify what ‘recovery’ is referring to. It’s still unclear what the pH trial duration was, and if there was a recovery phase like that in the oxygen trials.

Line 248 – is hypoxia survival significantly different between populations/latitudes? Please add details to text here, as well as Figure 2.

Line 248- Were females more tolerant than males in each population, under both low pH and oxygen? More details, including statistics, would be useful here. I suggest taking the pertinent info from the tables and adding to the text, and then the tables can be added to the SM.

Figure 3 caption says sex was important but not significant. What does that mean exactly? Additionally, why arrange panels based on collection and not latitude like Figure 2? It would be great to be consistent throughout. This also draws attention away from the latitudinal differences in hypoxia survival.

Line 277-280- information for the methods

Line 287: It would be useful to link the findings of this study to the extensive literature on the topic of diurnal flux in temp, pH and DO (Gobler has some good references in general, but I’m sure others exist for tide pools)

Line 323- Thermal tolerance is mentioned here, can the authors explain what this is referring to, what study, add details to this, etc.?

When referring to hypoxia or acid tolerance it would be better to just say the trait that is being assessed (i.e., survival) and eliminate any uncertainty as to what is being discussed in terms of ‘tolerance. ‘
