# Supplementary material for: Hypoxia tolerance, but not low pH tolerance, is associated with a latitudinal cline across populations of Tigriopus californicus
Source: PLoS One. 2022 Oct 27;17(10):e0276635. doi: 10.1371/journal.pone.0276635 (PMC9612455; doi:10.1371/journal.pone.0276635)
Supplement: Attachment — Submitted filename: Response to Reviewers.docx [file pone.0276635.s013.docx]

Editorial Comments

The manuscript received four reviews; all reviewers agreed that the manuscript is interesting and timely. Two reviewers recommended minor revisions (1 and 4), and two major revision (2 and 3). The reviews recommending minor revisions mostly focused on aspects that could strengthen the interpretation of the results and improve the quality of the discussion. In my view, these are excellent recommendations. The reviews recommending major revision reflect concerns on the quality/availability of the oxygen and pH data  and how they can bring into question the interpretation of the results. These are valid concerns.  If you have additional data that can put the concerns to rest, I encourage you to add them to the manuscript. Otherwise, the results may need to be reanalyzed and reinterpreted. At worse, the limitations of the experimental design and how they affect the results and their interpretation must be acknowledged. Reviewer 3 was also concerned about the validity of your correlations analysis.

Thank you for the time you took in reviewing our manuscript. Since we did not have additional data to supplement these experiments, we have clarified the style of the experiment in the introduction and methods sections and more explicitly identified the limitations in the discussion section.

When submitting your revision, we need you to address these additional requirements.

1. Please ensure that your manuscript meets PLOS ONE's style requirements, including those for file naming. The PLOS ONE style templates can be found at 
<https://journals.plos.org/plosone/s/file?id=wjVg/PLOSOne_formatting_sample_main_body.pdf> and 
<https://journals.plos.org/plosone/s/file?id=ba62/PLOSOne_formatting_sample_title_authors_affiliations.pdf>

We have updated the style to match the PLOS ONE requirements.

2. We note that you have stated that you will provide repository information for your data at acceptance. Should your manuscript be accepted for publication, we will hold it until you provide the relevant accession numbers or DOIs necessary to access your data. If you wish to make changes to your Data Availability statement, please describe these changes in your cover letter and we will update your Data Availability statement to reflect the information you provide.

We are ready to put our data on the Dryad repository once the article is accepted.

3. Please include your full ethics statement in the ‘Methods’ section of your manuscript file. In your statement, please include the full name of the IRB or ethics committee who approved or waived your study, as well as whether or not you obtained informed written or verbal consent. If consent was waived for your study, please include this information in your statement as well.

The UNC Institutional Animal Use and Care Committee only oversees vertebrate animal experiments. We did not need to apply, as we were working on an invertebrate system. We have added this clarification to the manuscript (lines 281-282).

Reviewer #1:

The manuscript covers a topic of great importance in the framework of impact of global change on marine organisms, and it would be very useful for those working in multiple-stressors, phenotypic plasticity, local adaptation, and the resilience of marine populations. However, I have some major concerns, regarding the, (i) theoretical background, (ii) experimental setup, and (iii) presentation of the results.

1. Page 3, Lines 40-44. These statements in the introduction are partially true, since the different behaviour of both gases, oxygen and CO2. While oxygen can increase during day time, CO2 cannot match with the same-interval due to differential diffusion and solubility of DO and CO2 can cause oxygen levels in seawater to come to equilibrium with prevailing atmospheric conditions more rapidly than CO2 (see Gobler & Baumann 2016). The resulting scenarios is a combination of scenarios, low pH/low O2, but also high O2 and low pH, due to the lag in the response of CO2.

We have added this reference to the introduction as well as an additional comment about the difference in diffusion rates in the introduction (lines 40-41). We also included additional references to oceanographic literature about pH and DO behavior to the discussion (line 370).

2. Authors do not present simultaneously in an orderly and graphic way the natural fluctuations in both pH and oxygen in the sampled rockpools. The variability of O2 and pH are presented separately, when precisely the focus of this study is to discuss the context of change of both variables. The sampling frequency is quite irregular, and of very low frequency, which also does not allow a good characterization of the environment; however, it would be very helpful if they could re-analyze the data and present it in a way consistent with the objectives of the study.

We have reformatted the figure (Fig 1) to make it easier to compare DO and pH rates both across and within locations as was recommended. We also consulted a statistician to reanalyze the measurements. Although there was nothing we could do about the frequency of sampling, we hope the new graphical layout facilitates a comparison of the variability of the locations. Finally, we moved the table to the supplemental information.

3. For the experimental setup, it is not possible to follow the experimental conditions during the incubation. What were the temperature, pH and O2 evolution during each experiment... Did the pH level affected during experiment of hipoxia?, same for the low pH conditions?

Temperature was kept constant (20°C) for all assays. Because pH levels increased during the hypoxia assays, we had run separate high pH assays as described in the methods (lines 183-188). The copepods were unaffected by high pH when oxygen was present, even though the pH was higher and the time period was longer than what they experienced in the hypoxia assay. We added a supplemental figure (S3 Fig) to show DO during the low pH assay. As explained in the methods, we do not believe low pH and hypoxia were confounded during the low pH assay (lines 219-223).

4. More importantly, the study did not consider measurements at night hours, exactly the time interval when lowest pH and O2 occurs in these environments. During night conditions oxygen can drop to almost zero in rocky tidal pools, pH lowest than 7.3 and and CO2 can increase up to > 3000 uatm (See Kwiatkowski et a. 2016). Since extreme levels in each rockypool along this cline play a disproportionately large role in shaping the physiology, ecology and evolution of these populations, I can see some part of this history is missed, and it is difficult for me to analyze any correlation with latitudinal patterns if we lack of extreme environmetnal conditions among studied populations.

We did not think it was safe for our observer to collect nighttime measurements given the locations of the pools, major health events of the observer at the time of the measurements, and the limitations of our equipment, which required the user to be present. We have added the reference to the discussion (line 376), and we more explicitly addressed the limitation of our methods in the discussion section (lines 374-378, 385-391). Thank you for the insightful comments.

Reviewer #2:

General Comments:
This manuscript looks at effects of body size, latitude (collection location), and sex on hypoxia tolerance and low pH tolerance in the copepod Tigriopus californicus. This paper examined how copepod cultures from six locations ranging from San Diego, CA to Friday Harbor Labs in Washington survive when exposed to acute exposures of low oxygen concentration and low pH. The authors hypothesized 1) that both stressors co-occuring would yield population-specific tolerance to either stressor, 2) that hypoxia and pH tolerance would inversely correlate with latitude, and 3) one of the evaluated effects would predict tolerance. The authors found that while latitude and length are predictors of performance with respect to hypoxia tolerance, only sex is a predictor for pH tolerance. Patterns of hypoxia tolerance with respect to latitude are converse to the authors expectations and may reflect duration of lab rearing rather availability of oxygen.
This is a very interesting study. I have few comments on the nature of this work. It is well designed, executed and analyzed. My only criticism would be the interpretation. There is nothing wrong with how the data is interpreted as it is, but it could benefit from more explanation or a deeper dive into why the authors believe they observed the patterns they did, particularly when considering the hypoxia tolerance pattern with latitude. I recommend the authors consider a few more ideas as to why hypoxia tolerance increases with latitude rather than decreases. Is there an evolutionary pattern that may be at play? Is the tolerance strictly plastic?

We have added more mechanisms of hypoxia and low pH tolerance to the discussion section (lines 401-409 and 412-415), and we added a possible relationship between the observed phenotypes and mitochondrial genetic patterns (lines 434-437). Finally, we elaborated on fitness consequences of sublethal stress with the caveat that our study did not investigate these other measures and are a recommended avenue of future research (lines 478-481).

Specific comments:
Lines 49-50, 308, 326-327: Fix references to match citation style.

Done.

Lines 204-206: “Importance” comes up a lot in this manuscript. How does one evaluate what the critical level of importance is? In other words, how important is “important”? Does a value >0.5 suggest that it is somehow not random?

We added a citation to a paper that discusses the use of importance as a statistical metric as well as some additional language we hope clarifies the use of this statistic (lines 273-275).

Table 2 – A figure depicting the temperature, DO, and pH changes across a day for each location would be easier to interpret. If possible, the authors should consider making one.

We revamped the supplemental figure that depicted DO and pH changes across locations, added a map of the locations, and moved it to the main manuscript (Fig 1).

Lines 249-250: “…although the latter was opposed by the effect of latitude.” What does it mean for one effect to oppose another? Is this meant to represent an antagonistic relationship?

Northern populations and older collections were more tolerant to hypoxia. However, since the northern populations also tended to be newer collections, the effect of latitude is opposite and therefore reduced by the effect of time since collection. We have rephrased this in the manuscript (lines 425-427).

Lines 310-315: So, hypoxia tolerance is a physical attribute and pH tolerance is a physiological one? This seems interesting. I suggest the authors discuss how both tolerance systems may involve physical and/or physiological mechanisms. For instance, the upregulation of oxidative stress proteins does involve certain reactive oxygen species that can be accumulated from hypoxia, not just low pH. The authors should expand on other stress tolerance mechanisms that are different for each of the stressors discussed in the manuscript.

We have included additional mechanisms to the discussion section as mentioned above.

Lines 324-335: This is very interesting and deserves more discussion. Please include more detail as to what the “temporal” correlations are. Are they across years? Or across the day?

Although temporal could refer to years, seasons, or time of day, we explored diurnal correlations. As such, we have clarified our meaning in the manuscript.

Reviewer #3:

Latitudinal cline in hypoxia tolerance does not result in correlated acid tolerance in Tigriopus californicus

This paper aims to determine if there is a correlation between low oxygen and low pH tolerance in populations of copepods from a broad latitudinal range. The topic is of great importance to the field and a better understanding of how the temporal variability of these two stressors impacts marine animals is needed. Including latitude and population-level responses is especially helpful, and difficult to do, making this contribution important. However, there are some issues with the current draft that should be addressed before publication. I hope my comments below are clear and helpful.

• The environmental data presented did not include continuous or night-time values. This is when you would expect the DO and pH minima to occur, thus linkages between the experimental design and field data are weak, and are the assumptions based on them. There isn’t much information about conditions as they relate to the latitudinal cline either.

As explained for Reviewer #1, we did not collect night time measurements so we elaborated on the limitations of our field data in the discussion section by reiterating their importance. We have also added references to previous research conducted on *T. californicus* pools in Canada which reported pH measures as low as 5.0 to provide additional support for our choice of target pH (lines 269-272).

• If I understood correctly, the low pH trials are confounded by co-occurring low O2. Even if the O2 levels were not at the experimental target (0.5 mg/L I think), 2 mg/L O2 during the low pH trials is still quite low (and hypoxic as this paper defines it!). To me, this makes it very difficult to say that there is ‘no evidence of correlation in tolerance for these two stressors’ Line 308. This should be discussed and addressed, and the correlation, and its interpretation, between the low O2 and pH trials should be reconsidered.

As explained to Reviewer #1, we provided more details about the DO levels during the pH trials in a graphical format and more details about our exploratory experiments. Although there were times when hypoxia and low pH were confounded in the low pH experiments, this was not true for the majority of trials, nor were low pH and low oxygen confounded for even a fifth of the time. Consequently, we do not feel these require revising our conclusions. However, we have added more explicit limitations to our interpretation of the results in the discussion section. Namely, we openly state that we only used one fitness measure to explore correlation in the traits, and that studies using other fitness measures may observe a different pattern (lines 597-599).

• Additionally, I found it misleading to discuss correlation between the low-O2 and low pH traits when they were not measured together (but separate single-stressor studies). I think that could be fixed by carefully fixing the text so that there is no confusion that a multiple stressor study was not performed, but single stressor trials. It also brings up the question of why the authors didn’t test these stressors together? There are many examples of multiple stressors (together) having very different, and important physiological impacts compared to single stressors.

We have added language throughout the manuscript to clarify the single-stressor nature of the studies. We also explained that our focus was on possible covariates for hypoxia and low pH tolerance, so we did not conduct co-stressor studies (lines 304-307). Ultimately, we have constraints on the amount of resources we could devote to such a study, but we agree that a co-stressor study would be highly valuable.

• It would be helpful if figures were reworked to help the reader to see the significant impact hypoxia and low pH (separately) have across populations/sites. A lot of the supplemental material was important and could/should be condensed and added to the main MS so the reader can more easily follow along with all the pertinent information readily available (e.g., a map that shows where the sites are and how they are environmentally distinct).

As also described for Reviewer #1, we have reworked these figures. We appreciate the feedback that we think significantly improved the interpretability of our data.

• It might be helpful to discuss the results from the low pH trial and the low oxygen trial separately and put less emphasis on the correlation analysis.

We rearranged the results section to discuss the low oxygen and low pH results separately before discussing the correlation analysis as recommended. We also explicitly connected our hypotheses to sections of the discussion in the same order as they appeared in the introduction. We hope this improves the clarity of our work, and we are grateful for the thorough feedback you have provided us.

Minor points:

Line 26 – both stressors when tested together? Will be helpful to differentiate single vs combined stress responses throughout the text

Done

Citation style format might be correct for journal, but seems to vary between numbers (13) and names (Edmands, 2018) throughout the text.

Corrected.

Line 57 add references for this. Also, specify that these multiple studies are (or are not) linking temperature, hypoxia and acid tolerance together as an adaptation. It’s confusing because I think you are trying to say that there are not studies on this topic (which I agree with and is the point you should emphasize -because your study is one of the first to test this). The first sentence of this paragraph doesn’t really say what I think you are trying to say here.

We have added references as well as reworked the first sentence to emphasize how our study is unique for exploring changes in hypoxia and low pH tolerance with latitude (lines 59-61).

‘Low pH’ is a better word to use than ‘acid’ – I would change this and be consistent throughout. As is, there is a mixture of terminology used.

Done

Also, define ‘hypoxia’ is border-line jargon without an exact definition, make sure this is defined once and used in that context throughout.

We reworded lines where we think our usage was confusing (lines 183-184).

Line 63- is Pc Pcrit?

P_crit_. We updated the symbol to be consistent with other PLOS One articles.

Line 88 – this is implies that both stressors are tested together, not singularly, this should be clarified

We clarified that this uses single-stressor studies here and throughout the manuscript.

Line 92-98- It would be helpful to add a map of all the locations, how far away from each other are these sites? The field measurement section here also could benefit from a figure showing the diurnal flux at the different sites, is it approx. the same magnitude along the gradient?

We have incorporated the map with the DO and pH measurements as indicated above to facilitate comparisons of variability with location.

The field data should be shown in the main text, maybe on the map, to highlight differences between sites

See previous statements.

Line 109- where these copepod cultures all being kept at the Bodega Marine Lab?

No, cultures and experiments were at UNC Chapel Hill. We added the location to the methods section (lines 148 and 174).

Line 114- up until now I thought the study was a multiple stressor study, not two single stressor studies. It would be helpful to ensure the experimental design is clear right away (in the intro). Also, was a multiple stressor (i.e., combined stressor) treatment attempted? If not, why? There can be different responses (synergistic, additive, antagonistic) when the stressors occur in combination, so some discussion on this missing element in the study would be beneficial.

As mentioned previously, we provided more explicit language in the introduction and methods section that these are single-stressor studies. Also, as stated above, we explained that we chose to explore multiple covariates of tolerance rather than focus on the species reaction to co-stress.

Table 1 could be converted into a map, and sent to the supplemental materials

Done

Line 117- where did experiments take place?

We added the location, UNC Chapel Hill, to the methods section.

Line 148- experimental design. It would be helpful if the authors briefly mentioned the experimental design earlier in the intro and methods. Also, please specify earlier what the target DO/pH levels were.

We clarified that these were single-stressor studies in the introduction and methods. We did not specify the target levels sooner because doing so disrupted the flow of the writing and provided reduced clarity of the methods, in our opinion. Instead, we used the term hypoxia more consistently and included a better justification for our target values by elaborating on the preliminary studies and including additional observations of their habitat variability.

How often were DO measurements were made during the trials (same for pH?), and what the variability in both pH and DO during these trials?

Although we sampled DO every 5 minutes but did not save the measurements so long as DO stayed below the 0.05 mg/L threshold. If DO exceeded 0.05 mg/L (such as occurred if the nitrogen supply ran out overnight), we removed the trial from our data (lines 194-199). We added a supplemental figure to show pH and DO variability during the low pH trial (S3 Fig), and these additional details have been added to the methods section (lines 217-223).

How was the oxygen kept stable?

Oxygen redissolved rapidly, so although we did not keep it a single value throughout the low pH assay, it also was not stressful during the assay.

It is intriguing to me that the pH went up during these trials, what was the starting pH? Why would this be?

We added to the methods that bubbling nitrogen to displace oxygen also displaces carbon dioxide (lines 183-184). However, additional experiments challenging the high pH tolerance of the copepods did not indicate that high pH was a stress for the copepods. See additional comments as described for Reviewer #1.

Line 152- can the authors elaborate on this experiment and how/why they chose the pH and DO values for the current study? It would be very helpful to be more specific about the exploratory experiments -this is interesting. – i.e., ‘75% of tested copepods were able to swim after xx hour exposure to oxygen concentrations of xx mg/L, therefore we chose to xx’

We have taken your advice and specified what level of survival we were targeting and how prior studies informed our decision for DO/pH level and length of exposure (lines 192-194 and 211-213).

The pH values seem very high to me. Is this normal?

See comments above.

Line 157- Please add the accuracy/precision of this DO probe and describe how it waw calibrated. Was this done with a 0-point calibration solution?

Done

Line 156- hypoxia was maintained for 20 hours- does this include the ~2 hours of ramping (when the nitrogen was turned on?)

We did not include the 2 hour of ramping as part of the 20 hours of exposure to hypoxia (lines 189-190). We hope the additional language in the methods as well as the supplemental figure (S2 Fig) clarify this.

Line 163- again, more details would be very helpful about the previous experiments

Done. Please see previous comments.

Line 161 – so the animals were exposed to 24 of low pH and 20 hours of low DO?

Yes. We clarified in the methods that we observed ~50% recovery at these time points.

Line 166- was this the same for the DO trial?

No. We hope the supplemental figure helps to clarify this distinction.

Line 182- how many copepods were not recovered? Why was this? Please put a number as to how many were lost. This is a concern if they were escaping from their cages.

Some copepods became permanently “stuck” in their cages, however this accounted for less than 3% of our sample. Additionally, no copepods migrated into other cages, or left their cages at all. We have elaborated on this in the methods (lines 242-245).

Figure S5 and S6- It would be useful to add time to these plots, to help identify what time of day you are referring to. Sunset and sunrise? What about noon? S6 is missing DO units. Also, would be good to have standard deviation for both of these to make comparisons easier.

We have added grey bars to indicate day and night, and we fixed the missing DO units (Fig 1). Both of these graphs (which are now a single figure) already include SD bars.

Line 219- again, it would be nice to have a map with this information in it, showing the variation in pH and DO in tidepools along the latitudinal gradient. It would be much easier to understand if Table 2, and Fig S5 and S6 were combined into one figure, in the main MS.

Done. See previous comments.

Line 231- what about night-time values? There are a lot of studies and data showing the pH and oxygen minima occur at night due to respiration. This is missing, and an important aspect to making claims such as this about DO, pH and latitudinal trends. Authors should take care in making interpretations from their measurements, or add additional data from other sources to help.

We do not have nighttime values for the reasons given to Reviewer 1. However, we have provided more language about the limitations of our data in the results and discussion sections.

Line 236 – Please clarify what ‘recovery’ is referring to. It’s still unclear what the pH trial duration was, and if there was a recovery phase like that in the oxygen trials.

We reworded some sections of the manuscript so that our use of the word recovery consistently means copepods were able to swim when prodded (e.g. they recovered from the stress) (line 412).

Line 248 – is hypoxia survival significantly different between populations/latitudes? Please add details to text here, as well as Figure 2.

Yes, there is a significant difference which we have added to the legend (Fig 2) as well as rephrased in the results (line 320).

Line 248- Were females more tolerant than males in each population, under both low pH and oxygen? More details, including statistics, would be useful here. I suggest taking the pertinent info from the tables and adding to the text, and then the tables can be added to the SM.

Males were more sensitive in 5 of the six populations for both hypoxia and low pH. Therefore, we specified this in the results (lines 321 and 346). We have also added the statistics to the results section as recommended and moved the tables to the supplemental information.

Figure 3 caption says sex was important but not significant. What does that mean exactly? Additionally, why arrange panels based on collection and not latitude like Figure 2? It would be great to be consistent throughout. This also draws attention away from the latitudinal differences in hypoxia survival.

We clarified that importance measures the frequency that a term appears in models and added a reference for using importance as a statistic (see comments for Reviewer #2). We also rearranged the panels to be organized by latitude instead of date of collection (Figs 3 and 4).

Line 277-280- information for the methods

We have moved the information to the methods section (line 272).

Line 287: It would be useful to link the findings of this study to the extensive literature on the topic of diurnal flux in temp, pH and DO (Gobler and Baumann has some good references in general, but I’m sure others exist for tide pools)

We added additional references, including the Gobler and Baumann study, to the first paragraph of the discussion. However, as we are not oceanographers, our field measurements are intended to raise interest rather than make a definitive statement about the physiochemical environment. We hope that future researchers will continue to include field measurements when studying organisms from this habitat.

Line 323- Thermal tolerance is mentioned here, can the authors explain what this is referring to, what study, add details to this, etc.?

This referred to studies mentioned in the introduction, and we added the references to the discussion as well (line 423).

When referring to hypoxia or acid tolerance it would be better to just say the trait that is being assessed (i.e., survival) and eliminate any uncertainty as to what is being discussed in terms of ‘tolerance. ‘

We have used the word survival more frequently as we think it makes sense for the clarity of the discussion section.

Reviewer #4:

Deconinck et al test for potential correlation in phenotypic response to two diurnally correlated stressors in six geographically distinct populations of Tigriopus californicus and the effect of sex, body size, and collection time on stress tolerance using a combination of laboratory based experiments and statistical modeling. The authors’ use of self-designed custom glove boxes to conduct their experiment is commendable. The paper is well suited for the audience of PLOS One. My comments largely focus on the experimental sections of this study, as computational modeling is not my area of expertise.

Abstract
The first sentence is a bit awkward – consider rephrasing.

We have rephrased the first sentence to clarify that stressors are covarying across the day.

Introduction
45-46 – This sentence needs a reference

Added

55-57 – This sentence needs a reference

Added

71 – You should cite Ligouri 2022 for an important counter-example to the other studies suggesting that low pH is stressful in copepods.

Liguori, Alyssa. "Multigenerational Life-History Responses to pH in Distinct Populations of the Copepod Tigriopus californicus." The Biological bulletin 242.2 (2022): 97-117.

Done. Thank you for the suggestion.

Although Table 2 does provide detailed records of field DO and pH conditions, it would be helpful to state the natural pH and DO range experienced by T. californicus in the first two paragraphs (Line 32 - 54) itself, especially for readers who might not be familiar with the study system.

We added the range of conditions reported in some of the literature as well as the references for those studies (lines 49-53).

Similarly, readers would find it easier to interpret sampling details (Table 1) and information from Table 2 if it was conveyed in a graphical manner (with locations plotted on a geographical map of the region), while retaining both the tables in the supplementary information section.

We have added a map to our environmental data and moved it to the main manuscript as recommended by other reviewers.

The introduction section could be strengthened by including examples of other species that show correlated phenotypic response to co-occurring stressors.

We added citations for other studies but did not include descriptions of the studies in the text (line 61). We feel that the studies in similar systems are more relevant than more distantly related systems and that there are sufficient examples in similar systems to demonstrate how the predictors we tested can influence hypoxia and low pH tolerance.

Methods:

Were copepods exposed to both stressors simultaneously or separately?

We have clarified the language in both the introduction and methods sections to convey that these are single stressor studies.

How was the exposure time chosen for hypoxia and low pH assays?

We included additional justification in the methods section as recommended by Reviewer #3 as well.

A picture of the custom glove-box chamber or its pictorial representation would be helpful to orient your readers to your unique experimental set-up.

A diagram of the glove box is available in the supplemental material IS1 Fig). We will also include the laser cutter blueprints with the data available on Dryad should the manuscript be accepted.

Results and Discussion:

The authors should consider justifying their choice of using survival as the sole fitness measures. The results and discussion sections also do not mention potential trade off mechanisms that could affect survival and other traits in an organism. For example, is it possible that the survivor animals from the stress trials had lower number of offsprings than the control animals? Relying on only one fitness measure also lends very little credit to line 367 as the authors are essentially using only one phenotypic measure to strike down the possibility of genetic covariance in their study system. I strongly recommend including more phenotypic measurements to support the claim in line 367.

We do not have additional phenotypic measures as we could not gather that data once some copepods (particularly those exposed to the low pH assay) were dead. We did include additional language around the limitations of our experimental design in the results and discussion sections, however as recommended by Reviewer #3.

238 – pH exposures are substantially lower than natural pH values for Tigriopus pools. Somewhere you need to better justify the use of such low pH values (besides the last paragraph of the discussion, which states that other studies have also done this…)

We added a reference to a prior observational study that reports lower pH values than the ones we measured as noted by Reviewer #3.
